# Assessment of autonomic symptoms may assist with early identification of mild cognitive impairment with Lewy bodies

**DOI:** 10.1002/gps.5703

**Published:** 2022-03-18

**Authors:** Calum A. Hamilton, James Frith, Paul C. Donaghy, Sally A. H. Barker, Rory Durcan, Sarah Lawley, Nicola Barnett, Michael Firbank, Gemma Roberts, John‐Paul Taylor, Louise M. Allan, John O’Brien, Alison J. Yarnall, Alan J. Thomas

**Affiliations:** ^1^ Translational and Clinical Research Institute Newcastle University Newcastle upon Tyne UK; ^2^ Population Health Sciences Institute Newcastle University Newcastle upon Tyne UK; ^3^ College of Medicine and Health Exeter University Exeter UK; ^4^ Department of Psychiatry University of Cambridge Addenbrooke's Hospital Cambridge UK

**Keywords:** autonomic symptoms, dementia with Lewy bodies, mild cognitive impairment

## Abstract

**Objectives:**

Autonomic symptoms are a common feature of the synucleinopathies, and may be a distinguishing feature of prodromal Lewy body disease. We aimed to assess whether the cognitive prodrome of dementia with Lewy bodies, mild cognitive impairment (MCI) with Lewy bodies (MCI‐LB), would have more severe reported autonomic symptoms than cognitively healthy older adults, with MCI due to Alzheimer's disease (MCI‐AD) also included for comparison. We also aimed to assess the utility of an autonomic symptom scale in differentiating MCI‐LB from MCI‐AD.

**Methods:**

Ninety‐three individuals with MCI and 33 healthy controls were assessed with the Composite Autonomic Symptom Score 31‐item scale (COMPASS). Mild cognitive impairment patients also underwent detailed clinical assessment and differential classification of MCI‐AD or MCI‐LB according to current consensus criteria. Differences in overall COMPASS score and individual symptom sub‐scales were assessed, controlling for age.

**Results:**

Age‐adjusted severity of overall autonomic symptomatology was greater in MCI‐LB (Ratio = 2.01, 95% CI: 1.37–2.96), with higher orthostatic intolerance and urinary symptom severity than controls, and greater risk of gastrointestinal and secretomotor symptoms. MCI‐AD did not have significantly higher autonomic symptom severity than controls overall. A cut‐off of 4/5 on the COMPASS was sensitive to MCI‐LB (92%) but not specific to this (42% specificity vs. MCI‐AD and 52% vs. healthy controls).

**Conclusions:**

Mild cognitive impairment with Lewy bodies had greater autonomic symptom severity than normal ageing and MCI‐AD, but such autonomic symptoms are not a specific finding. The COMPASS‐31 may therefore have value as a sensitive screening test for early‐stage Lewy body disease.

## Key points

- Autonomic symptoms are common in prodromal dementia with Lewy bodies (DLB) compared to normal ageing.
- Gastric and secretory symptoms are more commonly reported.
- Orthostatic and urinary symptoms are reported as more severe.
- Dysautonomia is a sensitive finding for prodromal DLB.

## 1 INTRODUCTION

The synucleinopathies, including Parkinson's disease (PD), dementia with Lewy bodies (DLB), and multiple system atrophy, are associated with pronounced dysfunction of the autonomic nervous system.^1^ Autonomic symptoms are therefore common in DLB and Parkinson's disease dementia (PDD).^2^ These may manifest in DLB prior to onset of dementia^3^ and so could be an early marker of a “bottom‐up” staged prodromal synucleinopathy.^4^ However, identifying autonomic symptoms is complicated by the range of causes of these in older adults, in particular these symptoms may occur in other forms of dementia such as Alzheimer's disease (AD),^5^ and so are not specific to DLB.^6^, ^7^

Recent efforts have led to the characterisation of the cognitive prodrome of DLB – mild cognitive impairment (MCI) with Lewy bodies (MCI‐LB) – in research settings, with autonomic symptoms included as supportive clinical features.^8^ While previous research has suggested that a retrospective history of autonomic dysfunction is common in DLB,^9^ that individuals with pure autonomic failure are at high risk of later conversion to DLB,^10^ MCI‐LB may feature greater heart rate variability than in AD,^11^ and that MCI‐LB may be characterised by abnormal cardiac metaiodobenzylguanidine (MIBG) imaging of the sympathetic nervous system,^12^ it is yet unclear whether MCI‐LB features greater severity of autonomic symptoms when prospectively assessed, and whether these may differentiate MCI‐LB from normal ageing or AD.

We therefore aimed to assess whether autonomic symptoms would be more severe in MCI‐LB than in normal ageing, and whether these might help to distinguish MCI‐LB from MCI due to Alzheimer's disease (MCI‐AD). We hypothesised that probable MCI‐LB would have more common and severe autonomic symptoms, assessed using the COMPASS (the Composite Autonomic Symptom Score scale), than cognitively healthy controls, and that the presence of autonomic symptoms would help differentiate MCI‐LB from MCI‐AD. We also hypothesised that cardiac MIBG abnormalities would be associated with greater autonomic symptom severity.

## 2 METHODS

### 2.1 Participants

Mild cognitive impairment patients and cognitively healthy controls were recruited into a longitudinal study, as described previously.^13^ To be eligible for either group, participants had to be aged 60 years or older and medically stable on study entry. The authors assert that all procedures contributing to this work comply with the ethical standards of the relevant national and institutional committees on human experimentation and with the Helsinki Declaration of 1975, as revised in 2008. All procedures involving human subjects were approved by the Newcastle and North Tyneside two Research Ethics Committee (15/NE/0420). Written informed consent was obtained from all subjects.

### 2.2 Mild cognitive impairment patients

Patients were recruited from local older persons' memory, psychiatric and neurology services in North East England given the presence of a health service diagnosis of MCI, and either the reported presence of any core clinical feature of DLB (complex visual hallucinations, rapid eye movement sleep behaviour disorder (RBD), cognitive fluctuations, or parkinsonism not preceding cognitive impairment by more than 12 months), or any supportive clinical feature of DLB (e.g. mood change or sleep disturbance). Exclusion criteria were the presence of dementia, no objective cognitive impairment, or possible clinical stroke, vascular cognitive impairment or frontotemporal aetiology.

### 2.3 Healthy controls

Cognitively healthy participants were recruited through the Join Dementia Research platform, and from friends or families of the patient group. They were required to be cognitively healthy with no MCI, dementia, or other suspected brain pathology, and normal structural MRI.

### 2.4 Design

All participants underwent detailed medical review and cognitive assessment,^13^ and imaging^12^, ^14^, ^15^ at baseline which have been previously reported, with further longitudinal review at approximately annual follow‐ups.

### 2.5 Clinical assessment, imaging, and differential classification

#### 2.5.1 Assessment

All participants were assessed by clinical interview with a clinical researcher for the medical reviews, with informant interview where available. This interview included the Composite Autonomic Symptom Score scale (COMPASS 31)^16^ which assessed six domains of autonomic dysfunction; orthostatic intolerance, vasomotor, secretomotor, pupillomotor, gastrointestinal and bladder symptoms. This scale contains up to 31 questions, and as few as 16 if the participant has not experienced any of the queried symptoms and responds negatively to the respective screening questions. The COMPASS was administered with the assistance of an informant when one was available.

#### 2.5.2 Imaging

Dopaminergic 123I‐N‐fluoropropyl‐2β‐carbomethoxy‐3β‐(4‐iodophenyl) single‐photon emission computed tomography (FP‐CIT) and cardiac MIBG imaging were carried out, as previously described.^12^, ^14^ FP‐CIT images were visually rated as normal or abnormal by a five‐person panel of experienced image analysts, blind to clinical information.^14^ MIBG images were classified as abnormal given a heart:mediastinum uptake ratio (HMR) of <1.86 based on data from locally‐recruited healthy controls.^17^ FP‐CIT and MIBG imaging results were then incorporated into differential classifications as reported below.

#### 2.5.3 Diagnosis

Research notes from this interview were independently assessed by an expert panel of old age psychiatrists (AJT, PCD, JPT) to judge both (1) the presence or absence of all‐cause MCI or dementia according to consensus criteria^18^, ^19^ and (2) the presence or absence of each of the four core clinical symptoms of MCI‐LB^8^: visual hallucinations, cognitive fluctuations, RBD, and parkinsonism. Following the research diagnostic criteria for MCI‐LB,^8^ a diagnosis of probable MCI‐LB was made if a patient had two or more core Lewy body symptoms or one core symptom in addition to a positive (abnormal) FP‐CIT or MIBG scan. Patients were diagnosed with possible MCI‐LB when they had only one core symptom or one abnormal scan (this group is included for information but following standard practice the primary comparisons were between probable MCI‐LB, controls and MCI‐AD). MCI‐AD was diagnosed following the criteria of Albert et al.^18^ Firstly, subjective and objective cognitive decline consistent with Alzheimer's disease were established, along with generally maintained independence of function in everyday life, and the absence of dementia. As directed in the criteria, other causes were then excluded including evidence of vascular cognitive impairment, primary progressive aphasia and behavioural variant frontotemporal dementia, along with Lewy body disease. We had access to participants' medical records and research MRI imaging, allowing us to identify and exclude cases of probable vascular cognitive impairment.

These diagnoses were updated at each annual follow‐up by the consensus panel. Following a clinical diagnosis of dementia and dementia subtype, participants did not receive any further follow‐up.

#### 2.5.4 Analysis

All analyses were conducted in *R* software. Group differences in the presence and severity of total and specific autonomic symptoms were assessed with zero‐adjusted gamma models to address two key issues likely to be introduced by the COMPASS scoring system: (1) the likely abundance of zero scores when symptoms are absent and (2) scores being additive and positive when symptoms are present leading to right‐skewness. This procedure allowed us to account for these two separate processes, symptom presence and symptom severity, in a single model by estimating two parameters: odds of non‐zero scores (*nu* parameter with logit link) and relative severity of non‐zero scores (*mu* parameter with log link). All models included age as a mean‐centred, scaled continuous covariate, and were estimated with the *gamlss* package. Simple associations were assessed with rank based Spearman's correlation tests.

As a small group with uncertain diagnosis, possible MCI‐LB were not considered in the primary hypothesis, but statistics are reported for additional context.

Receiver operating characteristic curves were plotted to assess the discriminatory utility of COMPASS total in identifying probable MCI‐LB. Diagnostic cut‐offs for discriminating probable MCI‐LB from MCI‐AD and healthy controls were identified by Youden's index.

#### 2.5.5 Data availability

Data supporting this analysis are available upon request through the Dementias platform UK (study reference: ‘SUPErB’).

## 3 RESULTS

### 3.1 Group differences

Clinical characteristics have been described previously in depth and are summarised in Table 1. No participants from any group were using midodrine or fludrocortisone to treat orthostatic hypotension. However, 28 MCI patients were receiving cholinesterase inhibitors, and 3 were receiving levodopa therapy. At the time of data locking, five MCI‐AD cases had developed dementia (all AD) and nine probable MCI‐LB had developed dementia (all probable DLB).

**TABLE 1:** Baseline characteristics of diagnostic groups

	Control (*N* = 34)	MCI‐AD (*N* = 38)	Possible MCI‐LB (*N* = 20)	Probable MCI‐LB (*N* = 41)	P value^a^
Age	74.2 (7.5)	75.1 (7.4)	73.8 (7.8)	74.2 (6.4)	0.664
Sex, n (%) female	10 (29%)	21 (55%)	9 (45%)	6 (15%)	<0.001
Mini mental state exam	28.5 (1.1)	26.8 (2.1)	26.0 (3.0)	26.6 (2.3)	0.892
Unified Parkinson's disease rating scale – Motor score	5 [0, 16]	10.5 [0, 62]	15 [1, 40]	21 [1, 50]	0.004
Instrumental activities of daily living	‐	8 [2, 8]	7 [3, 8]	6 [4, 8]	0.013
Clinical dementia rating	0 [0, 0]	0.5 [0.5, 0.5]	0.5 [0.5, 0.5]	0.5 [0, 0.5]	0.102
Cholinesterase inhibitor use	0 (0%)	6 (16%)	4 (20%)	18 (44%)	0.015
Levodopa use	0 (0%)	0 (0%)	0 (0%)	3 (7%)	0.088

*Note*: Mean (SD), Count (%), or Median [Range].

Abbreviation: MCI‐AD, mild cognitive impairment due to Alzheimer’s disease.

^a^ Probable MCI‐LB versus MCI‐AD asymptotic permutation test.

Of this cohort, 126 completed the COMPASS at baseline and seven did not; for this group, autonomic symptoms reported as present over the previous year are described in Table 2 along with age‐adjusted odds/odds ratios of any characteristics of that symptom being present (the *nu* parameter of the zero‐adjusted model, i.e. a symptom sub‐score >0). These indicated broad group differences in the presence of autonomic symptoms: MCI‐AD had significantly higher odds of gastrointestinal symptoms than in normal ageing, while MCI‐LB had significantly higher odds of secretomotor, gastrointestinal, and bladder symptoms.

**TABLE 2:** Autonomic symptoms reported by those who completed the COMPASS‐31 questionnaire, and odds/age‐adjusted odds ratios of these being present in comparison to cognitively healthy controls (inverted *nu* parameter from zero‐adjusted gamma model, exponentiated)

Symptom Reported	Control (*N* = 33)	MCI‐AD (*N* = 36)	Possible MCI‐LB (*N* = 19)	Probable MCI‐LB (*N* = 38)
Orthostatic intolerance	7 (21.2%)	8 (22.2%)	8 (42.1%)	16 (42.1%)
Odds of symptom presence ^b^	0.27 [0.11–0.59]	1.06 [0.33–3.41], 0.927	2.74 [0.80–9.79], 0.113	2.71 [0.97–8.18], 0.067
Vasomotor skin changes	4 (12.1%)	4 (11.1%)	2 (10.5%)	6 (15.8%)
Odds of symptom presence ^b^	0.14 [0.04–0.35]	0.89 [0.19–4.10], 0.881	0.89 [0.11–5.10], 0.896	1.37 [0.36–5.85], 0.649
Secretomotor symptoms				
Abnormal Sweating^a^	2 (6%)	7 (19.5%)	5 (26.4%)	11 (29%)
Dry Eyes	5 (15.2%)	7 (19.4%)	9 (47.4%)	7 (18.4%)
Dry Mouth	4 (12.1%)	8 (22.2%)	7 (36.8%)	15 (39.5%)
Odds of symptom presence ^b^	0.37 [0.16–0.77]	1.98 [0.72–5.68], 0.195	3.57 [1.09–12.37], 0.040	3.36 [1.25–9.56], 0.020
Gastrointestinal symptoms				
Decreased Appetite	8 (24.2%)	16 (44.4%)	6 (31.6%)	13 (34.2%)
Bloating After Meal	9 (27.3%)	7 (19.4%)	5 (26.3%)	12 (31.6%)
Vomiting After Meal	0 (0%)	2 (5.6%)	1 (5.3%)	3 (7.9%)
Cramping or Colicky Abdominal Pain	4 (12.1%)	5 (13.9%)	5 (26.4%)	9 (23.7%)
Bouts of Diarrhoea	7 (21.2%)	11 (30.6%)	10 (52.6%)	13 (34.2%)
Constipation	6 (18.2%)	12 (33.3%)	8 (42.1%)	16 (42.1%)
Odds of symptom presence ^b^	0.94 [0.47–1.88]	4.46 [1.57–13.77], 0.007	3.92 [1.14–16.10], 0.042	4.71 [1.67–14.46], 0.005
Bladder symptoms				
Lost Control of Bladder Function	5 (15.2%)	6 (16.7%)	7 (36.8%)	17 (44.7%)
Difficulty Passing Urine	3 (9.1%)	3 (8.3%)	3 (15.8%)	8 (21.1%)
Difficulty Completely Emptying Bladder	3 (9.1%)	8 (22.2%)	5 (26.3%)	21 (55.3%)
Odds of symptom presence ^b^	0.31 [0.13–0.67]	2.07 [0.73–6.17], 0.179	4.20 [1.27–14.93], 0.023	7.97 [2.83–24.69], <0.001
Pupillomotor symptoms				
Sensitivity to Bright Light	8 (24.2%)	8 (22.2%)	4 (21.1%)	13 (34.2%)
Trouble Focussing Eyes	1 (3.0%)	6 (16.7%)	5 (26.3%)	10 (26.3%)
Odds of symptom presence ^b^	0.37 [0.16–0.78]	1.36 [0.48–3.93], 0.563	1.88 [0.56–6.31], 0.306	2.17 [0.81–6.10], 0.133

Abbreviations: COMPASS‐31, Composite Autonomic Symptom Score scale 31; MCI‐AD, mild cognitive impairment due to Alzheimer’s disease.

^a^ Any decrease in sweating, or much more sweating than previously.

^b^ Baseline Odds [95% CI] for controls, age‐adjusted odds ratio [95% CI], *p* value for MCI groups.

The presence, severity, frequency, and any worsening of these symptoms contributed to symptom group sub‐scores, and a total autonomic symptom score. Medians and ranges for these scores are summarised in Table 3, alongside analysis of the *mu* component of the zero‐adjusted model: the relative severity of each symptom when present, and of overall autonomic symptom burden (compared to healthy controls in all cases). These indicated that overall age‐adjusted autonomic symptom burden was approximately twice as severe in MCI‐LB as in cognitively health comparators; an effect not found to be significant in MCI‐AD. While orthostatic intolerance was not significantly more common in MCI‐LB or MCI‐AD than in healthy controls (see Table 2), in both groups symptoms were rated as more severe than in controls. In addition to bladder symptoms being more common in MCI‐LB, they were also rated as significantly more severe when present.

**TABLE 3:** Autonomic symptom severity rated by the COMPASS‐31, and ratio coefficients of symptom severity when present (*mu* parameter from zero‐adjusted gamma model, exponentiated), in comparison to cognitively healthy controls

Median [Range] of symptom scores	Control (*N* = 33)	MCI‐AD (*N* = 36)	Possible MCI‐LB (*N* = 19)	Probable MCI‐LB (*N* = 38)
Composite Autonomic Symptom Score ‐ Total	4 [0, 18]	7 [0, 38]	9 [0, 48]	12.5 [0, 40]
Orthostatic Intolerance	0 [0, 4]	0 [0, 7]	0 [0, 7]	0 [0, 8]
Vasomotor	0 [0, 3]	0 [0, 4]	0 [0, 4]	0 [0, 4]
Secretomotor	0 [0, 3]	0 [0, 4]	1 [0, 5]	1 [0, 6]
Gastrointestinal	0 [0, 15]	2 [0, 19]	4 [0, 22]	4 [0, 17]
Bladder	0 [0, 3]	0 [0, 6]	1 [0, 8]	1 [0, 8]
Pupillomotor	0 [0, 6]	0 [0, 15]	0 [0, 15]	0 [0, 11]
Relative severity of symptoms present^a^				
Composite autonomic symptom score ‐ Total		1.47 [0.99–2.20], 0.061	2.17 [1.35–3.49], 0.002	2.01 [1.37–2.96], <0.001
Orthostatic intolerance		1.46 [1.10–1.95], 0.010	1.40 [1.04–1.89], 0.031	1.62 [1.27–2.08], <0.001
Vasomotor		0.90 [0.63–1.30], 0.583	1.36 [0.92–2.00], 0.129	1.10 [0.82–1.46], 0.522
Secretomotor		1.27 [0.87–1.84], 0.214	1.56 [1.04–2.36], 0.034	1.39 [0.98–1.98], 0.068
Gastrointestinal		1.03 [0.63–1.68], 0.902	1.37 [0.77–2.44], 0.279	1.25 [0.77–2.04], 0.361
Bladder		1.75 [0.99–3.11], 0.057	1.33 [0.72–2.46], 0.014	1.93 [1.15–3.25], <0.001
Pupillomotor		1.27 [0.80–2.02], 0.309	1.92 [1.16–3.17], 0.013	1.45 [0.94–2.23], <0.096

Abbreviations: COMPASS‐31, Composite Autonomic Symptom Score scale 31; MCI‐AD, mild cognitive impairment due to Alzheimer’s disease.

^a^ Age‐adjusted ratio [95% CI], *p* value.

Across both MCI groups, total COMPASS score was not significantly associated with global cognitive function (Addenbrooke's Cognitive Examination – Revised; rho = 0.10, *p* = 0.348), nor any specific domains of cognitive dysfunction (Addenbrooke's subscales: Attention/Orientation rho = 0.08, *p* = 0.469; Memory rho = 0.18, *p* = 0.082; Fluency rho = 0.01, *p* = 0.931; Language rho = −0.01, *p* = 0.900; Visuospatial rho = 0.09, *p* = 0.409) but was associated with reduced functional independence (Instrumental Activities of Daily Living; rho = −0.24, *p* = 0.030) and greater motor symptom severity (Unified Parkinson's Disease Rating Scale – Part III; rho = 0.32, *p* = 0.002).

A sensitivity analysis was conducted controlling for the use of cholinesterase inhibitors, since these might moderate the relationship between cognitive impairments and autonomic symptoms: use of cholinesterase inhibitors was associated with significantly lower total COMPASS and secretomotor symptom severities (95% CI: 19%–61% reduction in COMPASS symptom reported severity, 22%–53% reduction in secretomotor symptom reported severity).

When controlling for cholinesterase inhibitor use, which was less common in MCI‐AD (see Table 1), MCI‐AD was associated with significantly greater total autonomic symptom severity (1.66 times greater than controls [95% CI: 1.10–2.48]). This analysis did not meaningfully impact previous findings for probable MCI‐LB, which was still associated with significantly greater severity of total autonomic symptoms, orthostatic intolerance, secretomotor, and bladder symptoms. However, controlling for use of cholinesterase inhibitors also suggested that symptoms of orthostatic intolerance were not only more severe in this group but also significantly more common (Odds Ratio = 3.81, 95% CI: 1.20–12.08).

### 3.2 Secondary analysis: Metaiodobenzylguanidine imaging and autonomic dysfunction

An additional analysis was undertaken including both MCI‐LB groups only, to assess if MCI‐LB with abnormal MIBG imaging had more severe autonomic symptoms than those with normal MIBG, given that reduced cardiac uptake of MIBG may reflect a ‘body first’ rather than ‘brain first’ synucleinopathy,^20^ correlating with plasma alpha‐synuclein,^21^ and may therefore be associated with synuclein accumulation in the peripheral nervous system with resulting dysautonomia. There was no significant difference in overall COMPASS severity (*p* = 0.758), nor in orthostatic intolerance (*p* = 0.663), vasomotor (*p* = 0.865), secretomotor (*p* = 0.172), gastrointestinal (*p* = 0.276), bladder (*p* = 0.497), or pupillomotor (*p* = 0.705) sub‐scales.

In the overall cohort, there was a significant association between quantified MIBG Heart: Mediastinum ratio (HMR) and bladder dysfunction with lower HMR being associated with more severe bladder dysfunction (*p* = 0.017), but non‐significant associations with total COMPASS (*p* = 0.069), orthostatic intolerance (*p* = 0.403), vasomotor (*p* = 0.556), secretomotor (*p* = 0.234), gastrointestinal (*p* = 0.626), and pupillomotor (*p* = 0.133) symptoms.

### 3.3 Discriminating mild cognitive impairment with Lewy bodies from MCI‐AD and controls

**FIGURE 1:**
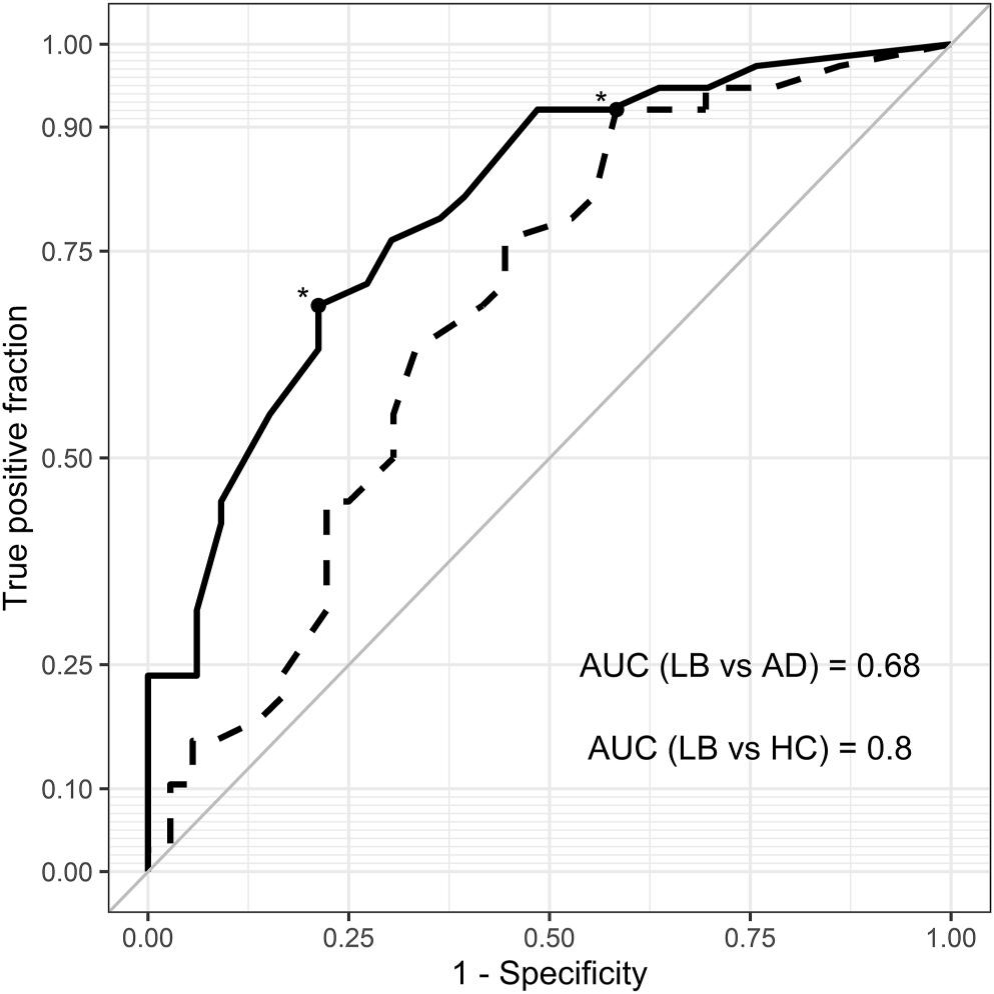
ROC curves for the discriminatory value of the Composite Autonomic Symptom Score scale 31 total score in identifying mild cognitive impairment with Lewy bodies (MCI‐LB) from mild cognitive impairment due to Alzheimer's disease (dashed line) and healthy controls (HC; solid line). Asterisks mark cut‐off values (4.5 and 9.5, respectively)

Receiver operating characteristic analyses found that total COMPASS score was a good discriminator of MCI‐LB from healthy controls, though less so in distinguishing probable MCI‐LB from MCI‐AD (see Figure 1). Youden's index identified the best cut‐off as >4.5 for the latter; this provided a high sensitivity of 92%, though specificity was low at 42%. In discriminating probable MCI‐LB from healthy controls, a cut‐off of >9.5 was identified, providing sensitivity of 68% and specificity of 79%; the previously identified cut‐off of >4.5 discriminated MCI‐LB from controls with low specificity of 52%.

### 3.4 Exploratory analysis with reduced COMPASS

An exploratory post hoc analysis was undertaken using a reduced COMPASS including orthostatic intolerance, bladder, and secretomotor symptom severity scores, based on broad group differences identified in the primary analysis. Receiver operating characteristic curves were plotted, and cut‐offs chosen which maximised Youden's index. In discriminating probable MCI‐LB from both MCI‐AD and healthy controls a >4.5 cut‐off was identified; this was much less sensitive to MCI‐LB than the full COMPASS‐31 (55% sensitivity), though specificity was substantially better, differentiating MCI‐LB from MCI‐AD with 78% specificity, and from controls with 94% specificity.

## 4 DISCUSSION

We hypothesised that, consistent with other clinical syndromes due to synucleinopathies, probable MCI‐LB would have greater autonomic symptom severity than cognitively healthy controls. The results supported this, identifying higher overall dysautonomia in probable MCI‐LB, but not in MCI‐AD who were intermediate to controls and MCI‐LB. MCI‐AD were more likely to experience gastrointestinal symptoms than controls, as were MCI‐LB, who were also more likely to experience secretomotor and bladder symptoms. When present, symptoms of orthostatic intolerance were more severe in MCI‐AD than in controls; this was also the case in MCI‐LB, in addition to more severe bladder symptoms. Composite autonomic symptom scores were highly sensitive to MCI‐LB, but specificity was low, consistent with relatively high rates of autonomic symptoms in other neurodegenerative aetiologies, as well as in cognitively healthy older adults.

The presence and severity of autonomic symptoms in probable MCI‐LB is consistent with evidence from other early clinical presentations of the synucleinopathies.^1^ These findings provide support for the inclusion of autonomic symptoms as a supportive clinical feature of MCI‐LB as in DLB^8^; autonomic symptoms appear to be common in the cognitive prodrome of DLB.

In a sensitivity analysis, we found some observational evidence that use of cholinesterase inhibitors may be associated with reduced presence or severity of some autonomic symptoms, and thereby obfuscate some group differences. This is consistent with recent evidence that cholinesterase inhibitors such as rivastigmine may have an antihypertensive effect in Parkinson's disease dementia, which may mediate the relationship between cholinergic therapies and cognitive outcomes.^22^ However, our study was neither designed nor powered to investigate these therapeutic effects: as with all such observational findings, these should be interpreted cautiously. Likewise, it remains possible that some symptoms observed may be side effects of medication to treat other (cognitive, clinical, or autonomic) symptoms.

We observed that autonomic symptoms were associated with greater motor impairment and reduced functional independence, though the direction of these relationships is unclear, and either or both could be bidirectional. Autonomic symptoms may impact on quality of life in MCI‐LB and may be amenable to treatment, with implications for patient care and management as in dementia.^2^, ^23^ These symptoms could contribute to risk of further complications; for example, orthostatic hypotension leads to heightened fall risks,^24^ and may also be prognostic of future decline.^25^

Autonomic symptom scales such as the COMPASS may be useful in specialist settings as relatively quick and simple screens for suspected MCI‐LB, particularly in research settings where more comprehensive assessment may be permitted to identify overlooked MCI‐LB, or in older adults' services where such data may be routinely collected. With high sensitivity to MCI‐LB at the >4.5 score cut‐off, absence of any autonomic symptoms may help to rule out MCI‐LB when other, specific clinical features are not observed. We explored the utility of an analysis of a reduced COMPASS measure targeting MCI‐LB (orthostatic intolerance, bladder, and secretomotor symptoms) as this may facilitate using autonomic assessments in clinical practice. We found that this had somewhat better specificity but with a high cost to sensitivity, and so could be useful as a briefer screen when specificity is desired. However, the presence of autonomic symptoms should not be taken as indicative of MCI‐LB in isolation, as there are multiple age‐related contributing factors^26^: for example, orthostatic hypotension is common in otherwise healthy older adults, being present in one in five older people,^27^ supporting the modest specificity of the COMPASS in this sample.

This study benefits from the inclusion of a thoroughly‐assessed, prospectively‐identified clinical cohort with comprehensive imaging and longitudinal medical review. However, due to the screening process seeking to identify potential Lewy body disease then autonomic symptoms could be more common in this MCI‐AD group than would be found in the wider population, which would mean that specificity might be higher than we found in this study. Both MCI‐AD and MCI‐LB groups are limited by the absence of specific AD biomarkers which might verify the presence of AD in the former, or identify mixed aetiologies in the latter.

These findings are limited by reliance upon self‐report of autonomic symptom presence and severity, which may be more sensitive to some symptoms than others; quantified measures of autonomic symptoms such as objective measures of orthostatic hypotension^28^ may provide clearer information on some symptoms into which patients may not have insight at the early stages of onset, as well as a method of quantifying any change over time. Relatedly, the COMPASS is likely not to have uniform sensitivity/specificity for each sub‐scale, as some symptoms may be more or less common in the general older population. This is evidenced by the variable presence and severities across different symptom scales in controls. Differences in autonomic symptom expression within MCI‐LB may therefore reflect differences in sub‐scale specificity, rather than any fundamental differences in the pathophysiology of different symptoms.

Cardiac scintigraphy with MIBG is a biomarker for DLB^29^ and in this cohort we recently reported that MIBG also had diagnostic accuracy in distinguishing MCI‐LB from MCI‐AD,^12^ providing further evidence that autonomic nervous system involvement is present at the MCI stage. However, we did not find any clear association between cardiac denervation assessed with MIBG imaging and autonomic symptoms within MCI‐LB. The number of subjects for this exploratory analysis was modest and any link between objective denervation and subjectively‐reported symptoms may be unclear at this early stage, particularly when autonomic symptoms may be mild and heterogeneous. Future research should incorporate longitudinal data to assess whether cardiac denervation anticipates later onset of subjectively‐reported or objectively‐measured dysautonomia, as discussed previously.

In conclusion, autonomic symptoms are more common and severe overall in MCI‐LB. High severity of autonomic symptoms is sensitive to MCI‐LB, but not specific to this condition.

## CONFLICT OF INTEREST

None.

## Data Availability

The data that support the findings of this study are available from the corresponding author upon reasonable request.
